# The Validation of Chinese Version of the Victimization Subscale of the Revised Peer Experiences Questionnaire

**DOI:** 10.3390/ijerph18062937

**Published:** 2021-03-13

**Authors:** Liheng Fan, Bu Liu, Zheng Jin, Xiangru Zhu

**Affiliations:** 1 Institute of Psychology and Behavior, Henan University, Kaifeng 475004, China; fanliheng@163.com; 2 Institute of Cognition, Brain and Health, Henan University, Kaifeng 475004, China; liubu36@sina.com; 3 International Joint Laboratory of Behavior and Cognitive Science, Zhengzhou Normal University, Zhengzhou 450044, China; jinzheng@zznu.edu.cn

**Keywords:** bullying, primary school, RPEQ, victimization, Chinese

## Abstract

In China, researchers have translated and validated several scales to measure victimization behavior. The aim of the present study was to validate the Chinese version of the victimization subscale of the Revised Peer Experiences Questionnaire (RPEQ) among primary school students. Primary school students aged between 8 and 13 years old (*n* = 1048) were asked to complete the Chinese version of the victimization subscale of the RPEQ and related scales. We examined internal consistency and the factor structure using confirmatory factor analysis (CFA). Depression, peer relationship, and sleep scales were used to measure construct validity. The CFA results suggested that the four-factor model had a good model fit. The results indicated that internal reliability was good (Cronbach’s α = 0.83). Construct validity was mostly supported by scores on the Chinese version of the victimization subscale of the RPEQ that strongly and positively correlated with depression and negatively correlated with peer relationship and sleep quality. The present study indicated that the Chinese version of the victimization subscale of the RPEQ has adequate reliability and validity for measuring bullying problems among Chinese primary school students.

## 1. Introduction

As children get older, peers become increasingly more important for children. Positive peer relationships provide children with important opportunities to express their feelings, improve thinking and language skills, and enhance many important social skills, such as empathy and cooperation [1]. Peer relationships can also contribute negatively to children’s social and emotional development through bullying, exclusion, and deviant peer processes [2]. Bullying is a form of aggression in which other children repeatedly and intentionally intimidate, harass, or physically harm a victim and is prevalent in many countries worldwide [3,4]. For example, a recent large population survey reported that the prevalence of bullying was ~30% among 12–17-year-old children in the United States [5]. In China, approximately 15% of middle school students were involved in regular interactions as either bullies or victims [6].

Self-rating bullying scales are widely used to assess the incidence and severity of specific bullying behaviors. The main advantage of a self-rating bullying scale is that it can be completed within minutes, and its applicationis relatively inexpensive. In English-speaking countries, the Olweus Bully/Victim Questionnaire (BVQ) [7,8] and Revised Peer Experiences Questionnaire (RPEQ) [9] are the most widely used scales for measuring school bullying. The BVQ includes two sets of items: one focuses on acts of victimization, while the other focuses on acts of bullying. The BVQ measures three types of bullying/victimization experiences: physical, verbal, and social. The Peer Experiences Questionnaire was developed by Vernberg, Jacobs, and Hershberger in 1999 [10], and Prinstein, Boergers, and Vernberg (2001) developed the RPEQ [9]. Consistent with the BVQ, the RPEQ also contains a victim version and an aggressor version. There are four subscales of each version: Overt Aggression/Victimization, Relational Aggression/Victimization, Reputational Aggression/Victimization, and Prosocial Behavior Toward Others/Receipt of Prosocial Behavior.

In 1999, Zhang, Wu, and Jones reported validation of the BVQ in Chinese journals [11]. The authors reported a primary school student version and a secondary school student version of the BVQ. Both versions have shown evidence of acceptable psychometric properties. Prof.Wenxin Zhang has been the main researcher on bullying in mainland China [6]. The Chinese version of the BVQ is widely used in Zhang’s research papers [12]. The Multidimensional Peer Victimization Scale (MPVS) is another internationally used scale to measure peer victimization [13]. In recent years, the Chinese version of the MPVS has been used by some Chinese researchers [14]. However, despite their regular use, the validity of the Chinese version of the BVQ and the Chinese version of the MPVS has not yet been tested. For the Chinese version of the BVQ, Zhang only provided reliability data and no structure data. His revision of the BVQ has not been further tested by other researchers. For the MPVS, Zhu and Lei translated the scale into Chinese but did not provide its internal structure or reliability data [15]. The MPVS has four factors, but Ji et al. (2011) only reported the internal structure and reliability data of two factors [14]. Therefore, given these drawbacks of the available Chinese bullying measurements, it is necessary to validate new measurement tools for use among Chinese students. Validation of the Chinese version of the RPVQ (victim version) will greatly facilitate the development of effective ways to assess school bullying in China.

For the victim version of the RPEQ [9], overt victimization involves being the target (by threats or in actuality) of physically aggressive behaviors, such as hitting, kicking, pushing, or chasing (i.e., “A teen chased me like he or she was really trying to hurt me”). Relational victimization includes being excluded from social events, activities, or conversations (i.e., “A teen did not invite me to a party or social event even though they knew that I wanted to go”). Reputational victimization refers to one’s social standing damaged by peer behaviors, such as rumor spreading and gossiping. Receiving prosocial behavior refers to the support from peers received by the victim (i.e., “Another teen helped me when I was having a problem”). Receipt of prosocial behavior can serve as a “buffer” or protective factor from the negative outcomes of peer victimization [16,17,18].

Compared with the BVQ and MPVS, the victim version of RPEQ includes the receipt of prosocial behavior, whereas the BVQ and MPVS do not include this dimension. This dimension is particularly useful for explaining the impact of peer victimization on individuals. An intriguing finding was that peer bullying can lead to internalization or externalization problems in behavior among one group, but they can lead to better academic achievement and higher peer acceptance among another group [16]. This result suggests that some factors modulate the relationship between peer aggression and individual adaptation. The receipt of prosocial behaviors from peers has been deemed a protective factor for victimized children [17]. For example, a previous study indicated that prosocial support from peers can modulate the relationship between peer victimization and loneliness [18]. The level of loneliness of a victim with social support is significantly lower than the level of loneliness of a victim without social support.

For school bullying, more research on middle school students has been conducted compared with primary school students. The prevalence of bullying among primary school students is not lower than it is among middle school students [19]. For example, in China, the prevalence of bullying in primary schools is approximately 25%, whereas the prevalence of bullying among middle school students is approximately 15% [20,21]. Previous studies indicated that students who are bullied in primary school tend to experience more health problems [22] and psychotic symptoms [23]. This demonstrates the importance of acknowledging that bullying victimization is an important problem. Therefore, it remains necessary to provide a validated instrument to evaluate peer victimization among Chinese primary school students.

The aim of the present study was to investigate the psychometric properties of the Chinese version of the RPEQ among primary school students, namely regarding its internal structure, internal consistency, and concurrent validity relative to other variables. We expected to find that the same four-factor structure would represent a good fit of the data of the victimization subscale of the RPEQ [24]. Two systematic reviews and meta-analyses have demonstrated that victims generally have a greater level of depression [25] and experience more sleeping problems [26]. With regard to validity relative to the other variables, we predicted that the victimization score on the RPEQ would be positively associated with depressive symptoms [27] and negatively associated with peer relationship and sleep quality [28,29].

## 2. Materials and Methods

### 2.1. Participants

The participants in the current study were 1048 primary school students (474 girls and 574 boys, 8–13 years old, M = 10.3 years, SD = 1.2 years) from two urban schools in Zhengzhou (ZZ), the provincial capital of Henan province, mainland China. Henan ranks 12th (about USD 8230) out of the 34 provinces in China in terms of GDP per capita, placing it a little below the national provincial average (about USD 10,000). Data were obtained by online survey and were collected in December 2020. We sent the survey link to the head teachers who then sent the link tothe parent’s mobile phone. The parent agreed to let the child fill out the questionnaire by handing the mobile phone to their child to complete the questionnaire. The head teacher of each class sent the survey link to 1225 parents. A total of 1048 students completed the survey. This study was performed in accordance with the Declaration of Helsinki and was approved by the Ethics Committee of the Henan University, China.

### 2.2. Measures

#### 2.2.1. RPEQ

We obtained the English version of the RPEQ (only the victimization part) from De Los Reyes and Prinstein’s paper [24]. The Chinese version of RPEQ was developed by using the back-translation method. The original RPEQ was translated into Chinese by the first and last authors of this manuscript. Then, we asked a bilingual translator from Brown University who is proficient in both English and Chinese to translate the Chinese version back into English. The authors compared the original English version with the back-translation and reconciled the differences to develop a single Chinese version of the RPEQ.

#### 2.2.2. PROMIS Depression

The Patient-Reported Outcomes Measurement Information System (PROMIS) is a National Institutes of Health funded initiative to develop and validate item banks and short forms for health outcomes across several domains, namely global health, physical function, fatigue, pain, sleep/wake function, emotional distress, and social health [30,31,32]. Item response theory (IRT) has been applied in developing and validating Patient-Reported Outcome Measurement Information System (PROMIS) item banks. In the present study, we used the PROMIS Pediatric Short Form v2.0—Depression 8b (eight items), which was provided by the PROMIS system (the Chinese version is not translated by the authors). This scale uses a 5-point Likert scale ranging from 1 (never) to 5 (always). The Cronbach’s α for the current sample was 0.90.

#### 2.2.3. PROMIS Peer Relationship

The PROMIS Peer Relationship Scale is an eight-item research tool designed to measure the perceived relationship with peers, using a 5-point Likert scale (1 = very strongly disagree, 5 = very strongly agree). There are no reverse-scored items, and higher scores indicate better peer relationships. The Chinese version of PROMIS Peer Relationship scale was obtained from the PROMIS system. In the present study, the Cronbach’s α was 0.91.

#### 2.2.4. Sleep

Three items from the Pittsburgh Sleep Quality Index [33] were used to assess sleep problems in children and adolescents. Students were asked to report sleep problems in the past month (i.e., “having trouble sleeping”, “having sleep disruption”, and “waking in the middle of the night or early morning”). All responses were on a 5-point scale ranging from 1 (never) to 5 (every day). The three items were summed to create an overall sleep problem score, with higher scores indicating more sleep problems. The three-item version sleep scale has been used in a previous study [34]. The Cronbach’s α of the three-item version Sleep scale in the present study was 0.81.

### 2.3. Statistical Analysis

#### 2.3.1. Internal Consistency Reliability

Internal consistency reliability is defined as the degree to which each item on the scale should be correlated with each other and the total score [35]. Cronbach’s α coefficient [36] is the most widely used index of internal consistency reliability. The Cronbach’s α was calculated to assess the internal consistency of the RPEQ.

#### 2.3.2. Confirmatory Factor Analysis

A confirmatory factor analysis (CFA)was performed on the RPEQ in the total sample. The model fit was evaluated by examining the root-mean-square error of approximation (RMSEA), the comparative fit index (CFI), the Tucker–Lewis index (TLI) and the standardized root-mean-square residual (SRMR). Regarding the RMSEA, values less than 0.05 are good, values between 0.05 and 0.08 are acceptable, values between 0.08 and 0.1 are marginal, and values greater than 0.1 are poor [37].TLI/CFI values above 0.90 and WRMR values less than 0.90 are indicative of good model fit [38].

#### 2.3.3. Construct Validity

Construct validity was evaluated by investigating the intercorrelations between the RPEQ and Depression, Peer Relationship, and Sleep Problem scales. We hypothesized that higher Depression and Sleep Problem scores would be associated with higher RPEQ scores, while the lower Peer Relationship scores would be associated with higher RPEQ scores.

#### 2.3.4. Moderation Analysis

The model 1 of Process macro (v 3.3) developed by Hayes [39] was used to test the moderating effect of receipt of prosocial behavior on the relationship between overt, relational, and reputational victimization and depression.

## 3. Results

### 3.1. Sample Characteristics

The total sample included *n* = 1048 children and adolescents. Table 1 shows the distribution according to grade and gender.

### 3.2. Scores of the Subscales of the RPEQ by Grade and Gender

Our aim in this section was to examine grade differences and gender differences in the prevalence of the total sample. Table 2 shows the distribution of the victims by grade level (Grades 3, 4, 5, and 6) for boys and girls.

### 3.3. Scores of the Four Dimensions of RPEQ by Gender and Grade

Gender effect: For overt victimization, univariate ANOVA revealed that the main effect of gender was significant (F (1,1040) = 14.97, *p* = 0.001, $\eta_{p}^{2}$ = 0.014). Boys received more overt aggression than girls. For relational victimization, the main effect of gender was also significant (F (1,1040) = 11.33, *p* = 0.001, $\eta_{p}^{2}$ = 0.011). Boys received more relational aggression than girls. For the receipt of prosocial behavior, the main effect was also significant (F (1,1040) = 15.45, *p* < 0.001, $\eta_{p}^{2}$ = 0.015). Boys also received more prosocial support from peers when being bullied. For reputational victimization, the main effect of gender was not significant (F (1,1040) = 1.07, *p* = 0.30).

Grade effect: For the overt victimization, univariate ANOVA indicated that the main effect of grade was marginally significant (F (1,1040) = 2.27, *p* = 0.08, $\eta_{p}^{2}$ = 0.007). The main effect of relational victimization was significant (F (1,1040) = 4.09, *p* = 0.007, $\eta_{p}^{2}$ = 0.012). A post hoc test (LSD) revealed that Grade 6 students received more overt aggression than students of the other three grades, but there was no significant difference between Grades 3, 4, and 5. For reputational victimization, the main effect was also significant (F (1,1040) = 11.17, *p* = 0.001, $\eta_{p}^{2}$ = 0.012). For the receipt of prosocial behaviors from peers, the main effect was also significant (F (1,1040) = 6.00, *p* = 0.001, $\eta_{p}^{2}$ = 0.017). For a specific comparison between the different grades, please see Table 3. For the four dimensions of the RPEQ, none of the interactions between gender and grade were significant, with all *p*-values being >0.1 (see Table 3).

### 3.4. Internal Consistency Reliability

For the present sample, the internal consistency analysis showed a Cronbach’s α of 0.83 for the RPEQ. Cronbach’s α values for Overt Victimization, Relational Victimization, Reputational Victimization, and Recipient of Prosocial Behavior subscales were 0.75, 0.71, 0.79, and 0.87, respectively.

### 3.5. Confirmatory Factor Analysis

The CFA for the present sample indicated that the four-factor model showed an acceptable model fit (RMSEA = 0.063; TLI = 0.94; CFI = 0.95; SRMR = 0.037). Item loadings on the four-factor solution for each version of the questionnaire were always significant (*p*< 0.001) and ranged from 0.614 to 0.811 for the victim version of RPEQ.

### 3.6. Construct Validity

For the present sample, the final scores (the sum of overt, relational and reputational victimization) on the RPEQ were positively correlated with scores on the Depression scale and the sleep score (*r* = 0.239, *p* < 0.001 and *r* = 0.350, *p* < 0.001) but negatively correlated with the peer relationship score (*r* = −0.178, *p* < 0.001). For the Overt Victimization subscale, the score was positively correlated with the depression score and the sleep score (*r* = 0.176, *p* < 0.001 and *r* = 0.266, *p*< 0.001) but negatively correlated with the peer relationship score (*r* = −0.137, *p* < 0.001). For the Relational Victimization subscale, the score was positively correlated with the depression score and the sleep score (*r* = 0.192, *p* < 0.001 and *r* = 0.309, *p* < 0.001) but negatively correlated with the peer relationship score (*r* = −0.172, *p* < 0.001). For the Reputational Victimization subscale, the score was positively correlated with the depression score and the sleep score (*r* = 0.240, *p* < 0.001 and *r* = 0.314, *p* < 0.001) but negatively correlated with the peer relationship score (*r* = −0.142, *p* < 0.001). For the Recipient of Prosocial Behavior subscale, the score was positively correlated with the sleep score and peer relationship score (*r* = 0.081, *p* = 0.009 and *r* = 0.274, *p*< 0.001) but not significantly correlated with the depression score (*r* = 0.055, *p* = 0.073) (see Table 4).

### 3.7. The Moderation Effect

We assessed whether the effects of victimization on depressive symptoms were moderated by the receipt of prosocial behaviors from peers. The results showed that the receipt of prosocial behavior hada significant moderating effect on the impact of overt victimization on depression (β = −0.104, *p* < 0.005). A further simple slope analysis found that, under high receipt of prosocial behavior (+1 *SD*), overt victimization has a significant positive predictive effect on depression (β = 0.134, *p* < 0.001), while under low receipt of prosocial behavior (−1 *SD*), overt victimization has a more significant positive predictive effect on depression (β = 0.342, *p* < 0.001); that is, the effect of overt victimization on depression decreased with the increase of receipt of prosocial behavior. The receipt of prosocial behavior also moderated the relationship between reputational victimization and depression (β = −0.131, *p* < 0.001). A further simple slope analysis showed that under high receipt of prosocial behavior (+1 *SD*), reputational victimization has a significant positive predictive effect on depression (β = 0.190, *p* < 0.001), while under low receipt of prosocial behavior (−1 *SD*), reputational victimization has a more significant positive predictive effect on depression (β = 0.451, *p* < 0.001); that is, the effect of reputational victimization on depression decreased with the increase of receipt of prosocial behavior. There was no modulation effect of receipt of prosocial behavior between relational victimization and depression.

We found that the effects of victimization upon sleep were not moderated by the receipt of prosocial behaviors from peers.

## 4. Discussion

In the present study, the results suggested generally good psychometric properties of the Chinese version of the RPEQ. The CFA results indicated a reasonable model fit of the four-factor modeling on the present sample. Construct validity was also supported in the present sample.

In the present study, Cronbach’s α values for Overt Victimization, Relational Victimization, Reputational Victimization, and Recipient of Prosocial Behavior were 0.75, 0.71, 0.79, and 0.87, respectively. A previous study that was conducted in the United States with 15–17-year-old students reported very similar results in terms of the Cronbach’s coefficient of the RPEQ subscales [24]. The CFA results were also acceptable. We found that RPEQ victimization scores negatively correlated with peer relationship and positively correlated with depression and sleeping problems. The correlation results provide evidence of good construct validity for the Chinese version of the victimization subscale of the RPEQ. The present results indicate that the Chinese version of the victimization subscale of the RPEQ has good psychometric properties for 8–13-year-old students in China.

Previous studies consistently found that boys are more likely than girls to experience overt victimization, but gender differences in relational victimization are inconsistent [40]. Researchers in China have also reported that primary school boys received more physical and relational aggression from peers than girls [41]. In the present study, boys reported more victimization of both overt and relational forms than did girls. For the receipt of prosocial behavior, boys also reported they received more peer support when being bullied than girls. There was no gender difference in reputational victimization between boys and girls.

Studies of samples from Western cultures and mainland China have fairly consistently reported that the prevalence of bullying behaviors decreases with age [21,42]. For example, Zhang (2002) found that both bullying and victimization steadily declined from Grade 2 to Grade 9 in China [21]. In this study, significant grade differences in the scores of three kinds of bullying behaviors were found among primary school students. For Grade 6 students, the scores of three kinds of bullying behaviors in primary school were significantly higher than they were for all the other grades. For Grade 5 students, only the score of reputational victimization was significantly higher than it was for students from Grade 3 and Grade 4. All the scores of the three kinds of peer victimization showed no significant differences between Grade 3 and Grade 4. In mainland China, parents generally have an overemphasis on academic success. Grade 6 students face greater stress to enter a good junior high school. Because students from good junior high schools are more likely to be admitted to good high schools, they are then more likely to be admitted to good universities. Graduating from a good university means higher social status and having a greater chance of finding a better job. Future research can explore how the academic stress of Grade 6 students affects peer victimization.

For the receipt of prosocial behavior, we found that girls but not boys who were bullied received less prosocial behavior from peers. This result is instructive for designing intervention strategies that target girl victims of bullying in primary school. Previous research has shown that the support of peers plays an important role in reducing the negative consequences of bullying [43]. However, girls in primary school received less support, suggesting that bullying interventions should focus more on programs to enhance peer support. For example, influential students should be encouraged to publicly oppose bullying behavior and encourage peers to help the victim of bullying behavior [44].

Peer victimization has been linked to various negative outcomes, and its connection to depression is especially strong [25,45]. A recent longitudinal study found that baseline peer victimization cannot predict the follow-up depression level but that baseline depression did predict subsequent peer victimization [46]. In the present study, all the scores of Overt, Relational, and Reputational Victimization were positively correlated with depression level. However, the correlation coefficients were about 0.2, which indicated that there are some mediators or moderators between peer victimization and depressive symptoms [47,48]. In the present study, we found that for victims of overt and reputational victimization, the receipt of prosocial behaviors from peers exerted a protective effect against the risk of depressive symptoms. Nonetheless, the receipt of prosocial behavior did not modulate the relationship between relational victimization and depressive symptoms. We speculate that relational bullying usually means that multiple classmates bully the victim, and overt and reputational bullying usually means that only one or two classmates bully the victim. This difference led to the fact that the receipt of prosocial behavior from other students did not alleviate the negative consequences of bullying behavior. This speculation, however, will require further confirmation.

In the present study, all the scores of Overt, Relational, and Reputational Victimization were positively correlated with sleeping problems. However, we found that the receipt of prosocial behaviors from peers can not moderate the relationship between peer victimization and sleeping problems. Previous studies generally found that sleeping problems may be a mediator of the association between peer victimization and negative outcomes [47]. Future studies can further explore whether other factors can moderate the relationship between peer victimization and sleeping problems.

### Limitations

The present psychometrice valuation has limitations. First, the RPEQ includes both a victimization scale and an aggression scale. Our study only validated the victimization subscale and not the aggression subscale. Second, the present study only obtained self-reported data on peer victimization. The accuracy of self-reported data was not tested by other sources, such as teachers and peers. Third, the subjects in this study were from one city in China. Data from more schools are needed to evaluate the generalizability of our results.

## 5. Conclusions

The Chinese version of the RPEQ had sufficient validity and was a reliable tool for assessing peer victimization in Chinese primary school students.

## Figures and Tables

**Table 1:** Distribution according to grade and gender.

	Grade 3	Grade 4	Grade 5	Grade 6
Boys	112	157	163	118
Girls	79	136	141	142
Total	191	293	304	260

**Table 2:** Scores of the three kinds of victimization and the receipt of prosocial behavior according to gender and grade.

		Boys	Girls
Grade 3 (*n* = 191)	Overt	3.44 ± 1.20	3.15 ± 0.53
	Relation	3.84 ± 1.39	3.34 ± 0.81
	Reputation	3.40 ± 1.22	3.18 ± 0.59
	Prosocial	9.65 ± 4.51	7.96 ± 3.81
Grade 4 (*n* = 293)	Overt	3.42 ± 1.13	3.21 ± 0.70
	Relation	3.96 ± 1.57	3.59 ± 1.35
	Reputation	3.47 ± 1.17	3.19 ± 0.67
	Prosocial	10.43 ± 4.93	9.16 ± 4.81
Grade 5 (*n* = 304)	Overt	3.36 ± 0.93	3.26 ± 0.90
	Relation	3.90 ± 1.39	3.72 ± 1.53
	Reputation	3.64 ± 1.43	3.74 ± 1.70
	Prosocial	10.94 ± 5.54	9.68 ± 5.20
Grade 6 (*n* = 260)	Overt	3.74 ± 1.72	3.29 ± 1.02
	Relation	4.25 ± 2.08	3.95 ± 1.99
	Reputation	3.92 ± 1.88	3.93 ± 1.82
	Prosocial	11.14 ± 5.06	10.36 ± 5.41

**Table 3:** The differences among the grades and the posthoc test results.

Grade	Grade	Overt Difference	*p*	Relation Difference	*p*	Reputation Difference	*p*	Receipt of Prosocial Difference	*p*
3	4	−0.02	0.98	−0.15	0.30	−0.03	0.80	−0.89	0.06
	5	−0.02	0.99	−0.19	0.20	−0.37 *	0.005	−1.39 *	0.03
	6	−0.22	0.04	−0.48 **	0.001	−0.61 **	0.001	−1.84 **	0.001
4	5	−0.002	0.99	−0.03	0.79	−0.33 *	0.004	−0.51	0.22
	6	−0.2	0.03	−0.33 *	0.01	−0.58 **	0.001	−0.95 *	0.03
5	6	−0.2	0.03	−0.3 *	0.03	−0.25 *	0.04	−0.44	0.29

* *p* < 0.05, ** *p* < 0.01.

**Table 4:** Correlations between scores on the Revised Peer Experience Questionnaire and other relevant variables.

Measure	Overt	Relation	Reputation	Prosocial	Depression	Sleep
Overt		0.55 **	0.64 **	0.18 **	0.18 **	0.27 **
Relation			0.59 **	0.20 **	0.19 **	0.31 **
Reputation				0.23 **	0.24 **	0.31 **
Prosocial					0.06 ^ns^	0.08 **
Depression						0.22 **

Note: ^ns^ nonsignificant. ** *p* < 0.01.

## Data Availability

The data presented in this study are available on request from the corresponding author.
